# Repetitive Transcranial Magnetic Stimulation (rTMS) Modulates Lipid Metabolism in Aging Adults

**DOI:** 10.3389/fnagi.2017.00334

**Published:** 2017-10-17

**Authors:** Weicong Ren, Jiang Ma, Juan Li, Zhijie Zhang, Mingwei Wang

**Affiliations:** ^1^Department of Psychology, Hebei Normal University, Shijiazhuang, China; ^2^Key Laboratory of Brain Aging and Cognitive Neuroscience of Hebei Province, Hebei Medical University, Shijiazhuang, China; ^3^Center on Aging Psychology, Key Laboratory of Mental Health, Institute of Psychology, Chinese Academy of Sciences, Beijing, China; ^4^Department of Rehabilitation, First Hospital of Shijiazhuang, Shijiazhuang, China; ^5^Department of Neurology, First Hospital of Hebei Medical University, Shijiazhuang, China

**Keywords:** rTMS, older adults, cardiovascular disease, lipid metabolism, HPT axis

## Abstract

Hyperlipidemia, one of the cardiovascular (CV) risk factors, is associated with an increase in the risk for dementia. Repetitive transcranial magnetic stimulation (rTMS) was applied over the right dorsolateral prefrontal cortex (DLPFC) to modulate serum lipid levels in older adults. Participants received 10 sessions of rTMS or sham stimulation intervention within 2 weeks. The serum lipid and thyroid hormone-related endocrine levels were assessed before and after the treatment. We found that rTMS significantly decreased serum lipid levels, including the total cholesterol (CHO) and triglyceride (TG); meanwhile, it also increased the thyroid-stimulating hormone (TSH) as well as thyroxine (T4) levels. This suggests that rTMS modulated the serum lipid metabolism by altering activity in the hypothalamo-pituitary-thyroid (HPT) axis. The trial was registered on the website of Chinese Clinical Trial Registry (http://www.chictr.org.cn).

## Introduction

Cardiovascular (CV) risk factors, such as hyperlipidemia, diabetes and hypertension, are associated with increased risk of dementia in older adults (Chuang et al., 2014). Longitudinal population-based studies have been used to assess the incidence of dementia in relation to CV diseases (CVD). Kloppenborg et al. (2008) reviewed the evidence for the association of CVD risk factors, including dyslipidemia, obesity, diabetes and hypertension with dementia. They found that these risk factors were indeed associated with an increased risk of dementia. Notably, for older adults, dyslipidemia appears to convey high risk of dementia.

Previous studies showed that cholesterol plays an important role in Alzheimer’s disease (AD) as it forms the core of neuritic plaques that characterize AD (Puglielli et al., 2003). Moreover, it has been suggested that blood lipids are promising AD biomarkers. For example, epidemiological studies proposed that high total serum cholesterol in midlife is linked to sporadic AD in old age (Notkola et al., 1998). Lipid measures, such as high-density lipoproteins (HDL) and total cholesterol (Kivipelto et al., 2006; Reitz et al., 2010), are currently used as assessment tools to evaluate the risk of AD and dementia (Wang H. L. et al., 2016). This suggests that vascular risk factors, especially blood lipids, should be regarded as a major target for preventive measures later in life.

It has been suggested that non-invasive brain stimulation (NIBS) is a promising therapeutic tool for CVD (Cogiamanian et al., 2010; Makovac et al., 2017). In a series of meta-analyses, it was demonstrated that NIBS, especially repetitive transcranial magnetic stimulation (rTMS), was effective in reducing the heart rate (HR) and enhancing the HR variability (HRV; Makovac et al., 2017), which are the risk factors for CVD.

George et al. (1996) applied rTMS over the prefrontal cortex (PFC) and found that stimulation of PFC was associated with increases in serum thyroid-stimulating hormone (TSH). Furthermore, in a case study, Trojak et al. (2011) applied rTMS to a patient with depression and demonstrated that serum TSH remained increased during the whole rTMS period. This suggests that rTMS may influence the hypothalamo-pituitary-thyroid (HPT) axis. In cross-sectional studies, serum TSH levels in the upper part of the reference range have been associated with low levels of HDL cholesterol (Boekholdt et al., 2010; Ittermann et al., 2012, 2013). In an 11-year prospective population-based study, it was demonstrated that high TSH levels within the reference range might be associated with decreased serum lipids (Åsvold et al., 2013). In animal studies, it was found that rTMS applied to aged mice could reverse the metabolic abnormalities of cholesterol levels (Wang et al., 2013). It can be speculated, from the above studies, that rTMS might alter the serum lipid level by modulating the HPT axis.

In the present study, we aimed to directly examine the aforementioned hypothesis. Specifically, we investigated the effect of rTMS on the serum lipid level, with rTMS applied over the right PFC. The total cholesterol (CHO), triglyceride (TG), high density lipoprotein cholesterol (HDL-C) and low density lipoprotein cholesterol (LDL-C) were measured as indexes of lipid level. Additionally, TSH, as well as thyroxine (T4), and triiodothyronine (T3), were assessed before and after the treatment. It is expected that lipid levels would be decreased after the session of rTMS treatment, along with alterations of thyroid-related hormones.

## Materials and Methods

### Participants

The participants met the following inclusion criteria: (1) age ≥60 years; (2) education ≥8 years; (3) a score of ≥21 on the Beijing Version of the Montreal cognitive assessment (MoCA; Yu et al., 2012); (4) right-handed; (5) a score of ≥2 on the Chinese memory symptoms scale (CMSS; Lam et al., 2005); (6) eligible for TMS procedures (Rossi et al., 2009). The exclusion criteria were: (1) history of neurological or psychiatric diseases; (2) history of brain damage; (3) history of thyroid disease; (4) dropout from the experiment because of bodily discomfort. All 30 elderly subjects, who were recruited and screened were randomly assigned into the rTMS group (*n* = 14) or the control (sham) group (*n* = 16) according to the random number table method.

This research is registered in the Chinese Clinical Trial Registry (ChiCTR-IOR-15006731). This study was carried out in accordance with the recommendations of the institutional review board of the Institute of Psychology, Chinese Academy of Sciences with written informed consent from all subjects. All subjects gave written informed consent in accordance with the Declaration of Helsinki. The protocol was approved by the review board of the First Hospital of Hebei Medical University, as well as the institutional review board of the Institute of Psychology, Chinese Academy of Sciences.

### Procedure

Participants were randomly assigned into the rTMS or control group. Both groups participated in rTMS or sham stimulation protocol for 2 weeks, including five sessions every week. The baseline assessment occurred the day before the first stimulation session. The post-intervention assessment occurred 1 day after the final stimulation session. All participants had blood drawn at 8:00 am. Blood samples were collected to detect blood lipids and thyroid hormones. Participants were kept blind to the study hypothesis.

### rTMS Protocol

The rTMS was applied with the MagPro X100 stimulator (MagVenture) and figure-of-eight coil (MFC-B65). The right dorsolateral PFC (DLPFC) was the target site, which was defined as the F4 region of the international 10-20 system for electroencephalography. The motor threshold was determined before the stimulation. It was defined as the minimum stimulator output value required to generate contraction of the abductor pollicis brevis for at least 5 of 10 consecutive pulses, and was measured via EMG by means of the Biopac MP100, using a contraction threshold of 50 mV (Moscatelli et al., 2016a,b,c). In each session, 10 Hz of rTMS was applied at 90% motor threshold to the stimulation location. In each stimulation block, rTMS pulses were present for 2 s and absent for 28 s; there were 40 blocks in total. Subjects in the sham stimulation group received the same stimulation protocol applied in the same manner, except that the coil was held at an angle of 90° (Kim et al., 2012).

### Output Measures

#### Hematological Examination

All subjects fasted prior to having blood drawn at 8:00 am before receiving rTMS intervention and the day after the intervention. Blood was drawn using right elbow flexion, routine disinfection and at about 10 ml for each patient. The venous blood was extracted into the coagulation vacuum tube and left at about 25°C for 1 h. The serum was separated with 3000 rotations/min centrifugal force (LODZ-1.2, Beijing centrifuge factory) for 10 min and was kept in a −70°C refrigerator. Before the experiment, serum samples were removed from the refrigerator and were re-dissolved in a water-bath at 37°C water. After the second centrifugation, supernatant was used to measure the indexes. Throughout the whole process, the instrument maintained a good working state, and the quality was controlled in strict accordance with the reagent manual for testing.

#### Blood Lipids

CHO, TG, HDL-C and LDL-C were measured using the enzyme method. The reagents were sealed away from light, stored at 2–8°C, and could not be inverted.

#### Thyroid-Related Hormones

T3, T4 and TSH were measured using the electrochemical luminescence method (Roche company cobas e601 automatic immunoassay). The reagents we used were: roche reagents T3 detection kit, Roche reagent T4 detection kit, and Roche reagent thyrotropin detection kit. Reagents were stored at 2–8°C away from light and not inverted.

**T3 measurement steps (competition law principle)**

1. Take 30 μl of the specimen; mix it with anti-T3 antibody (ruthenium mark) and 8-Anilino-1-naphthalenesulfonic acid (ANS) following the instructions; the ANS release combined T3.
2. Add streptavidin-coated microparticles and biotinylated T3. Biotinylated T3 occupies the relevant binding site on the labeled antibody (still in a free state).
3. Add the mixture to the measurement cell (immune complex), and adsorb the particles to the electrode through the magnet. Form the electrochemical luminescence by adding voltage to the electrode. Use the photomultiplier to detect T3 content.

**T4 measurement steps (competition law principle)**

1. Take 15 μl of the specimen; mix it with anti-T4 antibody (ruthenium) and ANS following the instructions; the ANS release combined T4.
2. Add streptavidin-coated microparticles and biotinylated T4. Biotinylated T4 occupies the relevant binding site on the labeled antibody (still in a free state).
3. Add the mixture to the measurement cell (immune complex), and adsorb the particles to the electrode through the magnet. Form the electrochemical luminescence by adding voltage to the electrode. Use the photomultiplier to detect T4 content.

**TSH assay procedure (double antibody sandwich principle)**

1. Take 50 μl of the sample; mix the anti-TSH monoclonal antibody (biotinylated antibody) and anti-TSH monoclonal antibody (ruthenium).
2. Add streptavidin-coated particles to the microparticles, and combine the mixture in step 1 with the microparticles. This combination uses the reaction between biotin and streptavidin.
3. Add the mixture to the measurement cell (immune complex), and adsorb the particles to the electrode through the magnet. Form the electrochemical luminescence by adding voltage to the electrode. Use the photomultiplier to detect TSH content.

### Data Analysis

The two-sample two-tailed Student’s *t*-test was used to assess the baseline characteristics (age, years of education, scores of CMSS and MoCA) of participants in both groups, and the Chi-squared test was used to assess the gender difference between group. Paired sample *t*-test was used to examine the effect of rTMS/sham stimulation on the serum lipid metabolism activity, as well as on the endocrine activity related to the thyroid. All statistical analyses were conducted using SPSS 19.0 (IBM Corporation, Somers, NY, USA). The absolute effect size, Cohen’s d (Cohen, 1988), were calculated to assess the effect of the rTMS intervention.

## Results

### Demographic Characteristics

No significant differences at baseline were found between the two groups in age, gender, education, memory complaint and MoCA (*p* > 0.05), as shown in Table 1.

**Table 1:** Characteristics of repetitive transcranial magnetic stimulation (rTMS) and sham groups.

Group	Age (years)	Gender		Education (years)	CMSS	MoCA
		M	F			
rTMS group (*n* = 14)	65.71 ± 5.08	3	11	12.21 ± 2.75	3.79 ± 1.81	28.07 ± 1.49
Sham group (*n* = 16)	66.62 ± 5.19	5	11	11.94 ± 3.04	4.25 ± 1.61	28 ± 2.07
*p*	>0.05^a^	>0.05^b^		>0.05^a^	>0.05^a^	>0.05^a^

*Note: CMSS, the Chinese Memory Symptoms Scale; MoCA, the Beijing Version of the Montreal cognitive assessment. ^a^The *p* value was obtained using a two-sample two-tailed *t* test. ^b^The *p* value was obtained using a two-tailed Pearson chi-square test. Data are shown as mean ± SD*.

### Effect of rTMS on Lipid Levels

The blood lipid levels between the two groups had no statistical difference at baseline (*p* > 0.05). As shown in Table 2, the CHO and TG levels were significantly lower after rTMS intervention (*p* < 0.05). Cohen’s d for CHO was 0.54, and that for TG was 0.31. While in the sham group, no significant differences were found between baseline and post-treatment assessments (*p* > 0.05).

**Table 2:** Comparison of lipid levels (mmol/L) between rTMS and sham groups.

Group	Test	CHO	TG	HDL-C	LDL-C
rTMS (*n* = 14)	Pre	6.23 ± 1.82	1.94 ± 1.49	1.43 ± 0.80	3.68 ± 0.79
	Post	5.37 ± 1.33	1.54 ± 1.10	1.19 ± 0.40	3.15 ± 1.28
	*t*	2.682*	2.513*	1.552	1.636
Sham (*n* = 16)	Pre	5.26 ± 1.25	1.45 ± 1.06	1.17 ± 0.33	3.25 ± 0.89
	Post	5.76 ± 1.70	1.68 ± 1.03	1.3 ± 0.52	3.48 ± 0.97
	*t*	−1.704	−1.706	−1.273	−1.519

Note: CHO, the total cholesterol; TG, triglyceride; HDL-C, high density lipoprotein cholesterol; LDL-C, low density lipoprotein cholesterol. *p < 0.05, two-tailed. Data are shown as mean ± SD.

### Effect of rTMS on Endocrine Activity Related to the Thyroid Gland

Serum thyroid hormone levels between the two groups had no statistical differences at baseline assessment (*p* > 0.05). As shown in Table 3, after rTMS intervention, the TSH and T4 levels were found to be significantly higher than those at baseline (*p* < 0.05). Cohen’s d for T4 was 0.40, and that for TSH was 0.27. In the sham group, no significant differences were found between pre- and post-assessments (*p* > 0.05).

**Table 3:** Comparison of thyroid hormone levels (nmol/L) between rTMS and sham groups.

Test	rTMS (*n* = 14)			Sham (*n* = 16)		
	T3	T4	TSH	T3	T4	TSH
Pre	1.65 ± 0.19	93.63 ± 14.24	3.45 ± 2.76	1.79 ± 0.25	102.53 ± 12.23	3.45 ± 2.35
Post	1.70 ± 0.25	99.14 ± 13.18	4.31 ± 3.56	1.75 ± 0.26	102.05 ± 10.55	3.32 ± 2.24
*t*	1.744	3.248**	2.379*	−0.428	−0.099	−0.181

Note: T3, triiodothyronine; T4, thyroxine; TSH, the thyroid-stimulating hormone. *p < 0.05, **p < 0.01, two-tailed. Data are shown as mean ± SD.

## Discussion

In this study, 10 Hz of rTMS was applied to healthy older adults with normal baseline lipid levels. After 10 sessions of stimulation, CHO and TG levels were significantly decreased, accompanied by increased TSH and T4, compared with the baseline condition, while no significant differences were found in the control group.

Our results indicate that rTMS may be effective for CVD risk factors. Previous studies confirmed the effect of rTMS on CV systems indexed by HR and HRV. In this study, we investigated the effect of rTMS on endocrine activity related to CVD risk factors and found that decreased serum lipid levels resulted from rTMS, suggesting rTMS influence on endocrine activity. In an animal study, Wang et al. (2013) explored the metabolic mechanism underlying the effects of rTMS. They observed that in mature mice, rTMS could reverse the metabolic abnormalities of cholesterol levels, to a degree similar to the young mice, showing that the rTMS could improve the metabolic profiles in PFC. Combining the present study with previous studies shows that rTMS could modulate the lipid metabolic activity associated with CVD.

The effect of rTMS on the serum lipid metabolic activity might result from its influence on the HPT axis. George et al. (1996) found that rTMS, applied over regions within the PFC, was associated with increases in serum TSH. Furthermore, Trojak et al. (2011) reported a significant increase in plasma TSH, above normal range, during low frequency rTMS (1 Hz) treatment. In addition, Osuch et al. (2009) demonstrated an increase in plasma T4 during treatment of anxiety disorders using low frequency rTMS. Thus, it can be inferred that rTMS may have significant effects on the pituitary-thyroid axis, which may potentially induce hyperthyroidism. Similar to previous studies, our study found significantly elevated TSH after rTMS, along with increased T4. Furthermore, in this study, we directly observed the alteration of serum lipid levels, suggesting that rTMS may influence the HPT axis and then affect the serum lipid levels.

Based on animal studies, we had confirmed that both normal aging (Wang et al., 2014) and pathological aging (Han et al., 2016; Wang J. et al., 2016; Shen et al., 2017) lead to metabolic abnormalities and that rTMS treatment could ameliorate metabolic abnormalities (Wang H. L. et al., 2016). For example, rTMS normalized prefrontal dysfunctions and cognitive-related metabolic profiling in aged mice (Wang H. L. et al., 2016). Besides, Lee et al. (2012) found that the effects of rTMS are related to changes in the brain lipids. In human studies, it also has been found that rTMS affects cortical metabolism (Bohning et al., 1999; Kimbrell et al., 2002). In the present study, we found that rTMS applied over the right DLPFC could alter the endocrine activity in normal aging adults. Combined evidence suggested that there is a pathway between the PFC and the hypothalamus, through which the PFC could modulate the endocrine activity.

As a non-invasive tool for the electrical stimulation of neural tissue (Barker et al., 1985), rTMS has the potential to modify excitability of the cerebral cortex at the stimulated site and at remote areas along functional anatomical connections (for a review, see Rossini et al., 2010). The PFC is linked to the thalamus (Alexander et al., 1986; Jones, 2007; Bolkan et al., 2017), and as a result, rTMS applied to the DLPFC might modulate the neural activity in the thalamus, which would then have an effect on the activity of the hypothalamus. The hypothalamus links the nervous system to the endocrine system, via the pituitary gland, in order to modulate the serum metabolic activity. It can be speculated that the PFC-thalamus pathway might play an important role in the present study to influence the HPT axis, and finally to modulate the serum metabolic activity of lipid levels.

Previous studies showed that CVD risk factors could be reduced with aerobic exercises. In a meta-analysis, Kodama et al. (2007) demonstrated that regular aerobic exercise could increase HDL-C level, which is associated with decreased risk of CVD (Maron, 2000). Alternative to physical exercise, in this study, the non-invasive rTMS is demonstrated as another promising tool for modulating CVD risk factors, specifically lipid levels, suggesting that rTMS is effective in minimizing CVD prevalence. Aerobic exercise may influence CV fitness, which results in changes to endocrine activity. While the effect of rTMS on endocrine activity might result from the altered excitability of the neuron, which may influence the signaling pathway of the HPT axis. This suggests that there is a pathway that underlies the transmission between electric signal and chemical signal. Future research on this issue is of great importance.

## Conclusion

rTMS of the PFC is associated with increases in the TSH and T4 levels and decreases in the serum CHO and TG levels. rTMS might alter the serum lipid levels by modulating the activity of the HPT axis. It is a promising tool for the modulation of lipid metabolism in older adults, and it reduces the risk for AD. In future studies, more indexes related to lipid metabolism and the activity of the HPT axis need to be assessed to clarify the effect of rTMS on the risk for CVD and to examine the direct relationship between lipid metabolism and the activity of the HPT axis following rTMS.

## Author Contributions

WR designed the work and drafted the manuscript. JM collected and analyzed the data. JL guided the research project. ZZ and MW revised the work and agreed to be accountable for all aspects of the work.

## Conflict of Interest Statement

The authors declare that the research was conducted in the absence of any commercial or financial relationships that could be construed as a potential conflict of interest.

## References

[B1] Alexander G. E. DeLong M. R. Strick P. L. (1986). Parallel organization of functionally segregated circuits linking basal ganglia and cortex. Annu. Rev. Neurosci. 9, 357–381. 10.1146/annurev.neuro.9.1.357 3085570

[B2] Åsvold B. O. Bjøro T. Vatten L. J. (2013). Associations of TSH levels within the reference range with future blood pressure and lipid concentrations: 11-year follow-up of the HUNT study. Eur. J. Endocrinol. 169, 73–82. 10.1530/EJE-13-0087 23641017

[B3] Barker A. T. Jalinous R. Freeston I. L. (1985). Non-invasive magnetic stimulation of human motor cortex. Lancet 325, 1106–1107. 10.1016/s0140-6736(85)92413-4 2860322

[B4] Boekholdt S. M. Titan S. M. Wiersinga W. M. Chatterjee K. Basart D. C. Luben R.. (2010). Initial thyroid status and cardiovascular risk factors: the EPIC-Norfolk prospective population study. Clin. Endocrinol. 72, 404–410. 10.1111/j.1365-2265.2009.03640.x 19486022

[B5] Bohning D. Shastri A. McConnell K. Nahas Z. Lorberbaum J. Roberts D.. (1999). A combined TMS/fMRI study of intensity-dependent TMS over motor cortex. Biol. Psychiatry 45, 385–394. 10.1016/s0006-3223(98)00368-0 10071706

[B6] Bolkan S. S. Stujenske J. M. Parnaudeau S. Spellman T. J. Rauffenbart C. Abbas A. I.. (2017). Thalamic projections sustain prefrontal activity during working memory maintenance. Nat. Neurosci. 20, 987–996. 10.1038/nn.4568 28481349 PMC5501395

[B7] Chuang Y. F. Eldreth D. Erickson K. I. Varma V. Harris G. Fried L. P.. (2014). Cardiovascular risks and brain function: a functional magnetic resonance imaging study of executive function in older adults. Neurobiol. Aging 35, 1396–1403. 10.1016/j.neurobiolaging.2013.12.008 24439485 PMC4282177

[B8] Cogiamanian F. Brunoni A. R. Boggio P. S. Fregni F. Ciocca M. Priori A. (2010). Non-invasive brain stimulation for the management of arterial hypertension. Med. Hypotheses 74, 332–336. 10.1016/j.mehy.2009.08.037 19775822

[B9] Cohen J. (1988). Statistical Power Analysis for the Behavioral Sciences. 2nd Edn. Hillsdale, NJ: Lawrence Earlbam Associates.

[B10] George M. S. Wassermann E. M. Williams W. A. Steppel J. Pascual-Leone A. Basser P.. (1996). Changes in mood and hormone levels after rapid-rate transcranial magnetic stimulation (rTMS) of the prefrontal cortex. J. Neuropsychiatry Clin. Neurosci. 8, 172–180. 10.1176/jnp.8.2.172 9081553

[B11] Han B. Yu L. Geng Y. Shen L. Wang H. Wang Y.. (2016). Chronic stress aggravates cognitive impairment and suppresses insulin associated signaling pathway in APP/PS1 mice. J. Alzheimers Dis. 53, 1539–1552. 10.3233/JAD-160189 27392857

[B12] Ittermann T. Thamm M. Wallaschofski H. Rettig R. Völzke H. (2012). Serum thyroid-stimulating hormone levels are associated with blood pressure in children and adolescents. J. Clin. Endocrinol. Metab. 97, 828–834. 10.1210/jc.2011-2768 22205713

[B13] Ittermann T. Tiller D. Meisinger C. Agger C. Nauck M. Rettig R.. (2013). High serum thyrotropin levels are associated with current but not with incident hypertension. Thyroid 23, 955–963. 10.1089/thy.2012.0626 23427935 PMC3752519

[B14] Jones E. G. (2007). The Thalamus. 2nd Edn. Cambridge, MA: Cambridge University Press.

[B15] Kim S. H. Han H. J. Ahn H. M. Kim S. A. Kim S. E. (2012). Effects of five daily high-frequency rTMS on Stroop task performance in aging individuals. Neurosci. Res. 74, 256–260. 10.1016/j.neures.2012.08.008 22974554

[B16] Kimbrell T. A. Dunn R. T. George M. S. Danielson A. L. Willis M. W. Repella J. D.. (2002). Left prefrontal-repetitive transcranial magnetic stimulation (rTMS) and regional cerebral glucose metabolism in normal volunteers. Psychiatry Res. 115, 101–113. 10.1016/s0925-4927(02)00041-0 12208488

[B17] Kivipelto M. Ngandu T. Laatikainen T. Winblad B. Soininen H. Tuomilehto J. (2006). Risk score for the prediction of dementia risk in 20 years among middle aged people: a longitudinal, population-based study. Lancet Neurol. 5, 735–741. 10.1016/s1474-4422(06)70537-3 16914401

[B18] Kloppenborg R. P. van den Berg E. Kappelle L. J. Biessels G. J. (2008). Diabetes and other vascular risk factors for dementia: which factor matters most? A systematic review. Eur. J. Pharmacol. 585, 97–108. 10.1016/j.ejphar.2008.02.049 18395201

[B19] Kodama S. Tanaka S. Saito K. Shu M. Sone Y. Onitake F.. (2007). Effect of aerobic exercise training on serum levels of high-density lipoprotein cholesterol: a meta-analysis. Arch. Int. Med. 167, 999–1008. 10.1001/archinte.167.10.999 17533202

[B20] Lam L. C. W. Lui V. W. C. Tam C. W. C. Chiu H. F. K. (2005). Subjective memory complaints in Chinese subjects with mild cognitive impairment and early Alzheimer’s disease. Int. J. Geriatr. Psychiatry 20, 876–882. 10.1002/gps.1370 16116581

[B21] Lee L. H. W. Tan C. H. Lo Y. L. Farooqui A. A. Shui G. Wenk M. R. (2012). Brain lipid changes after repetitive transcranial magnetic stimulation: potential links to therapeutic effects? Metabolomics 8, 19–33. 10.1007/s11306-011-0285-4

[B22] Makovac E. Thayer J. F. Ottaviani C. (2017). A meta-analysis of non-invasive brain stimulation and autonomic functioning: implications for brain-heart pathways to cardiovascular disease. Neurosci. Biobehav. Rev. 74, 330–341. 10.1016/j.neubiorev.2016.05.001 27185286

[B23] Maron D. J. (2000). The epidemiology of low levels of high-density lipoprotein cholesterol in patients with and without coronary artery disease. Am. J. Cardiol. 86, 11L–14L. 10.1016/s0002-9149(00)01462-4 11374848

[B24] Moscatelli F. Messina G. Valenzano A. Monda V. Viggiano A. Messina A.. (2016a). Functional assessment of corticospinal system excitability in karate athletes. PLoS One 11:e0155998. 10.1371/journal.pone.0155998 27218465 PMC4878742

[B25] Moscatelli F. Messina G. Valenzano A. Petito A. Triggiani A. I. Messina A.. (2016b). Differences in corticospinal system activity and reaction response between karate athletes and non-athletes. Neurol. Sci. 37, 1947–1953. 10.1007/s10072-016-2693-8 27544220

[B26] Moscatelli F. Valenzano A. Petito A. Triggiani A. I. Ciliberti M. A. P. Luongo L.. (2016c). Relationship between blood lactate and cortical excitability between taekwondo athletes and non-athletes after hand-grip exercise. Somatosensory Mot. Res. 33, 137–144. 10.1080/08990220.2016.1203305 27412765

[B27] Notkola I. L. Sulkava R. Pekkanen J. Erkinjuntti T. Ehnholm C. Kivinen P.. (1998). Serum total cholesterol, apolipoprotein E {FC12}e4 allele, and Alzheimer’s disease. Neuroepidemiology 17, 14–20. 10.1159/000026149 9549720

[B28] Osuch E. A. Benson B. E. Luckenbaugh D. A. Geraci M. Post R. M. McCann U. (2009). Repetitive TMS combined with exposure therapy for PTSD: a preliminary study. J. Anxiety Disord. 23, 54–59. 10.1016/j.janxdis.2008.03.015 18455908 PMC2693184

[B29] Puglielli L. Tanzi R. E. Kovacs D. M. (2003). Alzheimer’s disease: the cholesterol connection. Nat. Neurosci. 6, 345–351. 10.1038/nn0403-345 12658281

[B30] Reitz C. Tang M. Schupf N. Manly J. J. Mayeux R. Luchsinger J. A. (2010). A summary risk score for the prediction of alzheimer disease in elderly persons. Arch. Neurol. 67, 835–841. 10.1001/archneurol.2010.136 20625090 PMC3068839

[B31] Rossi S. Hallett M. Rossini P. M. Pascual-Leone A. (2009). Safety, ethical considerations, and application guidelines for the use of transcranial magnetic stimulation in clinical practice and research. Clin. Neurophysiol. 120, 2008–2039. 10.1016/j.clinph.2009.08.016 19833552 PMC3260536

[B32] Rossini P. M. Rossini L. Ferreri F. (2010). Brain-behavior relations: transcranial magnetic stimulation: a review. IEEE Eng. Med. Biol. Mag. 29, 84–96. 10.1109/MEMB.2009.935474 20176526

[B33] Shen L. Han B. Geng Y. Wang J. Wang Z. Wang M. (2017). Amelioration of cognitive impairments in APPswe/PS1dE9 mice is associated with metabolites alteration induced by total salvianolic acid. PLoS One 12:e0174763. 10.1371/journal.pone.0174763 28358909 PMC5373599

[B34] Trojak B. Chauvet-Gelinier J.-C. Vergès B. Bonin B. (2011). Significant increase in plasma thyroid-stimulating hormone during low-frequency repetitive transcranial magnetic stimulation. J. Neuropsychiatry Clin. Neurosci. 23:E12. 10.1176/appi.neuropsych.23.1.e12 21304111

[B36] Wang H. Geng Y. Han B. Qiang J. Li X. Sun M.. (2013). Repetitive transcranial magnetic stimulation applications normalized prefrontal dysfunctions and cognitive-related metabolic profiling in aged mice. PLoS One 8:e81482. 10.1371/journal.pone.0081482 24278445 PMC3838337

[B37] Wang H. Lian K. Han B. Wang Y. Kuo S. H. Geng Y.. (2014). Age-related alterations in the metabolic profile in the hippocampus of the senescence-accelerated mouse prone 8: a spontaneous Alzheimer’s disease mouse model. J. Alzheimers Dis. 39, 841–848. 10.3233/JAD-131463 24284365 PMC4532290

[B35] Wang H. L. Wang Y. Y. Liu X. G. Kuo S. H. Liu N. Song Q. Y.. (2016). Cholesterol, 24-hydroxycholesterol, and 27-hydroxycholesterol as surrogate biomarkers in cerebrospinal fluid in mild cognitive impairment and Alzheimer’s disease: a meta-analysis. J. Alzheimers Dis. 51, 45–55. 10.3233/JAD-150734 26836015 PMC6110663

[B38] Wang J. Yuan J. Pang J. Ma J. Han B. Geng Y.. (2016). Effects of chronic stress on cognition in male SAMP8 mice. Cell. Physiol. Biochem. 39, 1078–1086. 10.1159/000447816 27562628

[B50] Yu J. Li J. Huang X. (2012). The Beijing version of the montreal cognitive assessment as a brief screening tool for mild cognitive impairment: a community-based study. BMC Psychiatry 12:156. 10.1186/1471-244X-12-156 23009126 PMC3499377

